# RIP Kinase-Mediated Necrosis as an Alternative Mechanism of Photoreceptor Death

**DOI:** 10.18632/oncotarget.286

**Published:** 2011-06-10

**Authors:** Yusuke Murakami, Joan W. Miller, Demetrios G. Vavvas

**Affiliations:** ^1^ Retina Service, Angiogenesis Laboratory, Department of Ophthalmology, Massachusetts Eye and Ear Infirmary, Harvard Medical School, Boston, Massachusetts, 02114, USA

**Keywords:** Photoreceptor, necroptosis, receptor interacting protein kinase

## Abstract

Photoreceptor cell death is the terminal event in a variety of retinal disorders including age-related macular degeneration, retinitis pigmentosa, and retinal detachment. Apoptosis has been thought to be the major form of cell death in these diseases, however accumulating evidence suggests that another pathway, programmed necrosis is also important. Recent studies have shown that, when caspase pathways are blocked, receptor interacting protein (RIP) kinases promote necrosis and overcome apoptosis inhibition. Therefore, targeting of both caspase and RIP kinase pathways are required for effective photoreceptor protection. Here, we summarize the current knowledge of RIP kinase-mediated necrotic signaling and its contribution to photoreceptor death.

## INTRODUCTION

**Figure 1:**
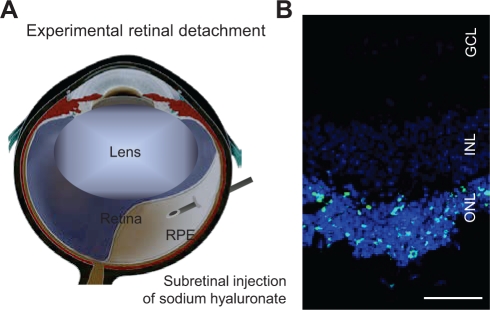
Experimental retinal detachment **A.** Scheme of experimental retinal detachment. Subretinal injection of sodium hyaluronate causes physical separation of photoreceptors from the underlying RPE. **B.** TUNEL staining 3 days after experimental retinal detachment. TUNEL positive cells are detected in the outer nuclear layer (ONL). INL: inner nuclear layer; GCL: ganglion cell layer; scale, 50 μm.

Photoreceptor death is the ultimate cause of vision loss in many retinal disorders. Photoreceptors die when they are physically separated from the underlying retinal pigment epithelium (RPE) and choroidal vessels, which provide metabolic support to the outer layers of the retina. Retinal detachment occurs in various retinal disorders, including age-related macular degeneration (AMD) [1], diabetic retinopathy [2], as well as rhegmatogenous retinal detachment. Although surgery is carried out to reattach the retina, only two-fifths of patients with rhegmatogenous retinal detachment involving the macula recover 20/40 or better vision. Histological examination of the retina in experimental retinal detachment, which is created by subretinal injection of sodium hyaluronate in animal eyes, showed that photoreceptor death is first identified at 12 hours, peaked by around 3 days, and dropped to a low level by 7 days after retinal detachment (Fig. 1A and B) [3,4,5]. Interestingly, the retina in patients with rhegmatogenous retinal detachment exhibits a similar pattern and time course of photoreceptor death observed in experimental retinal detachment [6]. These studies suggest that photoreceptor death may be one of the causes of vision loss after retinal detachment.

AMD is the most common cause of adult blindness in the western world [7]. Severe vision loss in late stage AMD results from choroidal neovascularization (called wet or neovascular AMD) or geographic atrophy (called dry AMD) [8]. In wet AMD, choroidal neovascular vessels leak serous or hemorrhagic fluid, causing detachment of RPE or photoreceptors, subretinal or intraretinal hemorrhage, and consequent fibrovascular scarring. Although anti-vascular endothelial growth factor (VEGF) therapies have shown visual improvement in many patients with neovascular AMD [9,10,11], some patients still do not respond to these therapies and 2/3 of patients do not have visual improvement. Because photoreceptor loss underlies the pathology of AMD [12], neuroprotective agents targeting photoreceptor death may be used in combination with anti-VEGF therapies to improve visual outcomes. In dry AMD, geographic atrophy is a serious cause of vision loss. It results from a slowly progressive atrophy of RPE and photoreceptors. Histological studies of geographic atrophy have suggested that RPE cells die first, followed by degeneration of photoreceptors [1,13]. On the other hand, macular translocation studies have shown that RPE atrophy recurs under the translocated macula after surgery, suggesting the possibility that photoreceptors may cause RPE degeneration in geographic atrophy or that RPE cells are impaired in handling the metabolic/trophic demands of the macular photoreceptors. [14,15,16].

In other retinal degenerative disorders such as retinitis pigmentosa, photoreceptor death is the basis for visual decline [17]. Retinitis pigmentosa is a group of inherited retinal disorders, affecting over 1 million individuals worldwide. Although genetic analyses have identified over 40 different genetic mutations [Retinal Information Network (RetNet) at *http://www.sph.uth.tmc.edu/Retnet/*], the mechanisms by which these mutations cause photoreceptor death are unclear and these diseases remain intractable [18,19]. Therefore, identification of the mechanisms involved in photoreceptor death is critical to developing new treatment strategies for these retinal disorders associated with photoreceptor loss.

## TWO DISTINCT FORMS OF CELL DEATH: APOPTOSIS AND NECROSIS

Apoptosis and necrosis are two distinct modes of cell death defined by morphological appearance [20]. In 1972, Kerr, Wyllie and Currie used the Greek term ‘apoptosis’ (dropping off of petals from plants) to describe a specific morphological aspect of cell death [21]. Apoptosis is accompanied by rounding-up of the cell, reduction of cellular volume, chromatin condensation, and engulfment by resident phagocyte. Apoptosis is the best-characterized type of programmed cell death, and these morphological changes are largely mediated by the activation of caspase family of cysteine proteases [22]. In contrast, ‘necrosis’ (from the Greek, dead) is associated with a gain in cell volume, swelling of organelles, plasma membrane rupture and subsequent release of intracellular contents with ensuing inflammation. Previously, necrosis has been considered a passive, unregulated form of cell death, but recent evidence indicates that some necrosis can be induced by regulated signal transduction pathways such as those mediated by RIP kinases [23]. This programmed form of necrosis is termed programmed necrosis or necroptosis [24,25].

## METHODS TO DETECT APOPTOSIS AND NECROSIS

Although several biochemical methods to detect cell death have been developed, there is no perfect method that can specifically discriminate between apoptosis and necrosis. For example, whereas detection of phosphatidylserine exposure is known as a marker of early apoptosis, necrotic cells also externalize phosphatidylserine before membrane permeabilization in some cells [26]. TUNEL staining, which was initially thought to detect specifically apoptotic cells, also labels DNA breaks in necrotic cells [27,28]. Conversely, cell impermeable dye such as propidium iodide, which is used to label necrotic cells, also detects late-stage apoptosis. Biochemical detection of key molecular events in apoptosis (e.g. caspase cleavage) and necrosis (e.g. RIP kinase phosphorylation) or inhibition of these molecules by pharmacological or genetic approaches may provide significant information for the specific roles of each cell death mode. However, it should be noted that these molecular pathways are not completely independent and may cross-talk with each other especially in the late phase of cell death. Although transmission electron microscopy has been used less frequently over the past decade, it is still one of the most sensitive and direct methods to detect morphological changes in cell death [29,30]. Given all of these findings, it is apparent that a combination of several distinct techniques is needed for the proper classification of cell death modalities.

## CASPASE SIGNALING

Cystein aspartate-specific proteases or caspases are the central molecules involved in initiation and execution of apoptosis [22]. There are at least 7 mammalian caspases that have an important role in apoptosis, and they are divided into two major classes: the initiator caspases, caspase-2, -8, -9 and -10; and the effector caspases, caspase-3, -6, and -7. The initiator caspases cleave inactive forms of effector caspases, thereby activating them. Once activated, the effector caspases cleave a broad spectrum of protein substrates, which in turn lead to induction of apoptosis.

**Figure 2:**
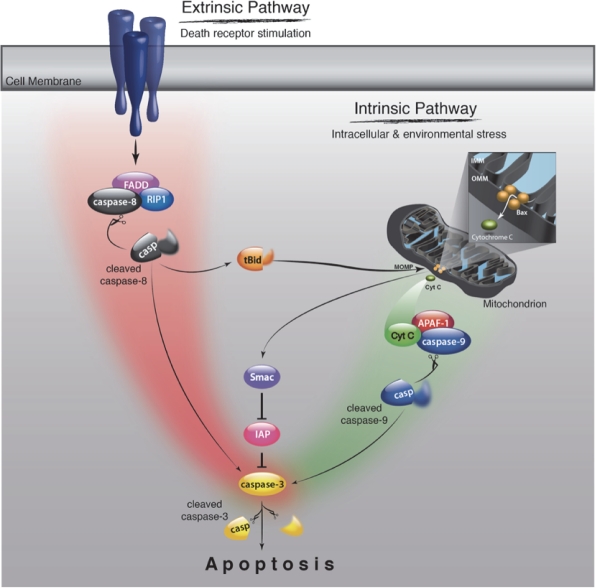
Schematic of caspase signaling pathway The extrinsic pathway is initiated by biding of death ligands such as TNF-α and Fas ligand to their cell-surface death receptors such as TNFR and Fas. The death domains of these receptors recruit adaptor molecules like FADD and caspase-8, which leads to the activation of caspase-8. Activated caspase-8 cleaves the effector caspases such as caspase-3, thereby activating them and inducing apoptosis. The extrinsic pathway interacts with the intrinsic pathway via caspse-8-mediated cleavage of Bid. The intrinsic pathway is initiated by release of mitochondrial intermembrane proteins such as cytochrome *c* and Smac/Diablo into the cytosol. Released cytochrome *c* forms an apoptosome with Apaf-1 and caspase-9, which leads to caspase-9 activation. Smac/Diablo enhances caspase activation through the neutralization of IAP proteins.

Caspase activation occurs mainly through the extrinsic and intrinsic pathways [31] (Fig. 2). The extrinsic pathway is initiated by binding of extracellular death ligands such as TNF-α and Fas ligand to their cell-surface death receptors like TNFR and Fas [32]. The death domains of these receptors recruit adaptor molecules like Fas-associated death domain (FADD) and caspase-8, forming the death inducing signaling complex (DISC) [33]. The formation of DISC leads to activation of caspase-8, which in turn mediates cleavage of effector caspases. The extrinsic pathway can cross-talk with the intrinsic pathway through caspase-8-mediated cleavage of Bid, a BH3-only member of the Bcl-2 family proteins [34,35]. Bid cleavage releases a truncated fragment that triggers the release of mitochondrial proteins, thereby initiating intrinsic caspase cascade as described below.

The intrinsic pathway is mediated by mitochondria [36]. In response to intracellular and environmental stress, mitochondria release intermembrane proteins such as cytochrome *c* and second mitochondria-derived activator of caspases (Smac)/direct inhibitor of apoptosis-binding protein with low pI (Diablo) into the cytosol. Released cytochrome *c* triggers the formation of an apoptosome along with apoptotic protease activating factor-1 (Apaf-1) and caspase-9 in the presence of ATP, which leads to caspase-9 activation [37]. Smac/Diablo enhances caspase activation through the neutralization of inhibitor of apoptosis (IAP) proteins [38,39].

## THE ROLE OF CASPASES IN PHOTORECEPTOR DEATH

There is no doubt that caspases play a central role in the induction of apoptosis especially in the early stages; however, accumulating evidence suggests that the caspase pathway may not be the sole mediator of neuronal cell death in pathological conditions. In experimental models of retinal detachment, although enzymatic activities of caspase-8, -9, -3, and -7 increase in the retina after retinal detachment [5,40], caspase inhibition by a pan-caspase inhibitor fails to prevent photoreceptor loss [4]. Reduced expression of Apaf-1 in *forebrain overgrowth* mutant mice exhibits partial, but not complete, protection against photoreceptor death after retinal detachment [41]. There is conflicting evidence regarding caspase activation during photoreceptor death in inherited retinal degeneration. Whereas several studies reported an increased activity of caspase-3 and -8 in a model of inherited retinal degeneration (rd1 mice), others showed that activation of caspase-9, -8, -7, -3, and -2 is not observed in rd1 mice [42] and that caspase inhibition by the pan-caspase inhibitor Z-VAD or testing in mice deficient in caspase-3 is not sufficient to prevent photoreceptor loss [43,44]. Intraperitoneal injection of a caspase-3 inhibitor provides mild and transient protection with no effect after 13 days of age in rd1 mice [45].

In the mature brain and retina, it has been demonstrated that caspase-dependent apoptosis is down-regulated because of a differentiation-associated reduction in Apaf-1 and caspase-3 expression and increased efficacy of IAPs [46,47,48]. Segura and others reported that the long form of the Fas apoptotic inhibitory molecule is predominantly expressed in neurons and prevents the activation of caspase-8 induced by Fas [49]. Gene expression profiling of the retina after retinal detachment and in inherited retinal degeneration revealed changes in multiple cell death pathways as well as caspase signaling [50,51]. Recent studies have shown that several caspase-independent inducers of cell death such as apoptosis-inducing factor (AIF), calpains, and poly(ADP-ribose) polymerases 1 (PARP-1) are activated during retinal degeneration [44,52,53]. These findings indicate the involvement of multiple death signaling mechanisms in photoreceptor death, and suggest that inhibition of the caspase pathway alone may not be sufficient to prevent photoreceptor loss in retinal degenerative disorders.

## CLINICAL STUDIES USING CASPASE INHIBITORS

There are only a few clinical trials employing caspase inhibitors in human diseases (http://clinicaltrials.gov/). PF-03491390 (formally called IDN-6556) is an anti-apoptotic caspase inhibitor that has advanced into phase 2 clinical trials [54]. PF-03491390 is an irreversible and broad-spectrum caspase inhibitor, and blocks the activities of caspase-1, -2, -3, -6, -7, -8, and -9 [55]. In phase 1 and 2 studies, intravenous or oral administration of PF-03491390 was generally well tolerated [56,57,58]. Oral administration of PF-03491390 significantly reduced serum AST and ALT in a phase 2 study for patients with chronic hepatitis C virus [57]. Larger clinical studies are needed to establish the safety and efficacy of caspase inhibitors. There has been no caspase inhibitor-based clinical study for retinal and neurodegenerative disorders [59].

## EVIDENCE OF NECROSIS IN PHOTORECEPTOR LOSS

Although most of studies have focused on apoptosis as a mechanism of photoreceptor death, previous morphological analyses demonstrated the presence of photoreceptor necrosis as well as apoptosis after retinal detachment and retinal photic injury [60,61]. Interestingly, Arimura and others showed that the vitreous level of high-mobility group box 1 (HMGB1) is increased in human eyes with retinal detachment [62]. HMGB1 is a nuclear DNA-binding protein, which is mainly present in the nucleus and is passively released into the extracellular space from necrotic cells [63]. These findings suggest that necrosis and subsequent release of intracellular content may occur in human retinal degeneration. Furthermore, using experimental models of retinal detachment, we recently demonstrated via electron microscopy and molecular biology analysis that programmed necrosis is a significant mode of photoreceptor cell death after RD and that the RIP kinase pathway plays an important role in the induction of photoreceptor necrosis, especially when caspase pathways are inhibited [64]. Rosenbaum and others also reported that RIP kinase inhibition by RIP1 kinase inhibitor protects retinal neuronal cells against retinal ischemic-reperfusion injury [65]. Thus, these results suggest that not only apoptosis but also necrosis are important for cell death during retinal degeneration, and that targeting necrosis signaling may be a novel therapeutic strategy for treatment of retinal disorders.

## RIP KINASE SIGNALING

### RIP1 polyubiquitination mediates pro-survival NF-κB activation

**Figure 3:**
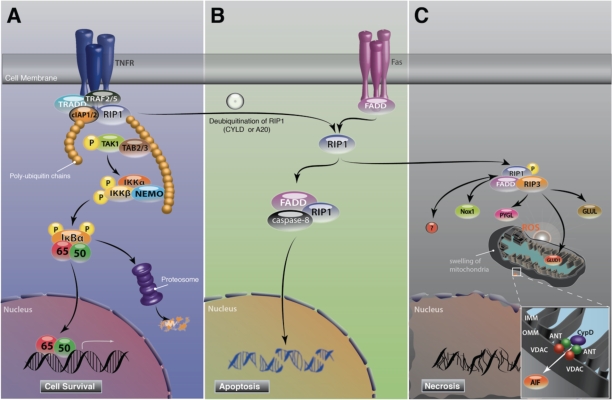
Schematic of RIP signaling pathway **A.** In response to TNF-α stimulation, RIP1 is recruited to TNFR and forms a membrane associated complex I with TRADD, TRAF2/5 and cIAP1/2, which in turn leads to polyubiquitination of RIP1 and pro-survival NF-κB activation. **B.** RIP1 switches function to a regulator of cell death when RIP1 is unubiquitinated by A20 or CYLD. Deubiquitination of RIP1 leads to the formation of cytosolic DISC with FADD and caspase-8, the so-called complex II. In contrast to TNF signaling, Fas stimulation directly forms DISC. Activation of caspase-8 in DISC leads to apoptosis induction. During apoptosis, RIP1 is cleaved and inactivated by caspase-8. **C.** In conditions where caspases are blocked or cannot be activated efficiently, RIP1 binds to RIP3, and both RIP1 and RIP3 kinases are phosphorylated at RIP1-RIP3 complex. RIP1 kinase phosphorylation is critical for necrosis induction. In response to TNF-α, RIP1 binds to NADPH oxidase 1 and produces superoxide. Activated RIP3 binds to PYGL, GLUL and GLUD1 and increases the production of mitochondrial ROS. ROS overproduction leads to mitochondrial dysfunction, resulting in the release of mitochondrial pro-death proteins.

RIP1 is an adaptor protein that acts downstream of death domain receptors and is essential for both cell survival and death [66]. RIP1 consists of an N-terminal serine/threonine kinase domain, an intermediate domain, a RIP homotypic interaction motif (RHIM), and a C-terminal death domain. After TNF-α stimulation, RIP1 is recruited to TNFR and forms a membrane associated complex I with TNF receptor-associate death domain (TRADD), TNF receptor-associated factor 2 or 5 (TRAF2/5) and cellular IAP1 or 2 (cIAP1/2), which in turn leads to polyubiquitination of RIP1 [67,68]. This polyubiquitin chain serves as an assembly site for transforming growth factor-β-activated kinase-1 (TAK1), TAK1 binding protein 2 or 3 (TAB2/3) and inhibitor κB kinase (IKK) complex, and mediates pro-survival NF-κB activation (Fig. 3A)[69]. Cells deficient for both cIAP1 and cIAP2, in which RIP1 polyubiquitination and NF-κB activation are blunted, are sensitized to TNF-mediated cell death [68,70]. Consistent with these results, RIP1 knockout mice die soon after birth with reduced NF-κB activation and extensive apoptosis in lymphoid and adipose tissues [71].

### RIP1 kinase phosphorylation at the crossroad of apoptosis and necrosis

RIP1 switches its function to a regulator of cell death when it is deubiquitinated by A20 or cylindromatosis (CYLD) [72,73]. Deubiquitination of RIP1 abolishes its ability to activate NF-κB after TNF-α stimulation, and leads to the formation of cytosolic DISC with FADD and caspase-8, the so-called complex II [67]. As described above in *caspase signaling*, DISC formation leads to caspase-8 activation and subsequent apoptosis. In contrast to TNF signaling, Fas directly recruits RIP1, FADD and caspase-8 to the plasma membrane and forms DISC (Fig. 3B) [74,75,76]. During apoptosis, RIP1 is cleaved and inactivated by caspases [77].

Although many cell lines are protected against death receptor-induced apoptosis by a pan-caspase inhibitor, Vercammen and others found that, in mouse L929 fibrosarcoma cells, caspase inhibition does not prevent TNF- or Fas-induced cell death and the cells acquire a necrotic morphology [78,79]. In 2000, Holler and others discovered that RIP1 kinase is a key molecule that induces necrotic cell death mediated by death receptors [80]. Recently, three independent studies have identified that the interaction of RIP1 and RIP3 through their RHIM domain is a critical step for the phosphorylation of their kinase domains and subsequent necrosis (Fig. 3C) [81,82,83]. The complex containing RIP1 and RIP3 is termed the necrosome. Although RIP1 is expressed ubiquitously in all cell types, RIP3 expression levels differ amongst cells and tissue [82,84]. Interestingly, He and others showed that death receptor-mediated necrosis correlates with RIP3 expression levels [82]. Gene knockout or knockdown of RIP3 completely inhibits RIP1 kinase phosphorylation and subsequent necrosis after death receptor stimulation [81]. These findings indicate that RIP3 is a key regulator of RIP1 kinase phosphorylation and necrotic signaling.

In 2005, Degterev, Yuan, and others using chemical library screening, identified small compounds named necrostatins that specifically inhibit death receptor-mediated necrosis [25]. Necrostatins have been shown to specifically inhibit RIP1 kinase phosphorylation during necrosis without affecting death receptor-induced NF-κB activation [85]. RIP1 kinase activity appears to be important for necrosome formation, as necrostatin-1 abolishes the formation of the RIP1-RIP3 complex and RIP3 kinase phosphorylation during necrosis [81,82]. Cho and others propose that another unknown kinase activated by RIP1 may mediate RIP3 phosphorylation, based on the findings that ectopically expressed RIP1 does not phosphorylate RIP3 [81]. The activities of RIP1 and RIP3 may be mutually regulated in a necrosome signaling complex.

## THE ROLE OF RIP KINASE IN PHOTORECEPTOR DEATH

**Figure 4:**
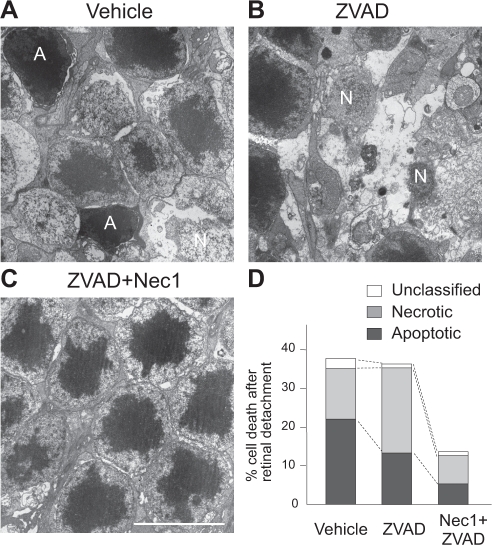
Caspase inhibition exacerbates necrosis after retinal detachment **A-C.** TEM photomicrographs of photoreceptors 3 days after retinal detachment in the retina treated with vehicle (*A*), pan-capase inhibitor Z-VAD (*B*), or Nec-1+Z-VAD (*C*). A: apoptotic cell. N: Necrotic cell. Scale bar, 5 μm. Photoreceptors showing cellular shrinkage and nuclear condensation were defined as apoptotic cells, while photoreceptors associated with cellular and organelle swelling and discontinuities in plasma and nuclear membrane were defined as necrotic cells. Electron-dense granular materials were labeled simply as end-stage cell death/unclassified. **D.** Quantification of apoptotic and necrotic photoreceptor death after retinal detachment. Z-VAD treatment decreased apoptosis but exacerbates necrosis. Nec-1+Z-VAD significantly suppressed necrotic photoreceptor death.

**Figure 5:**
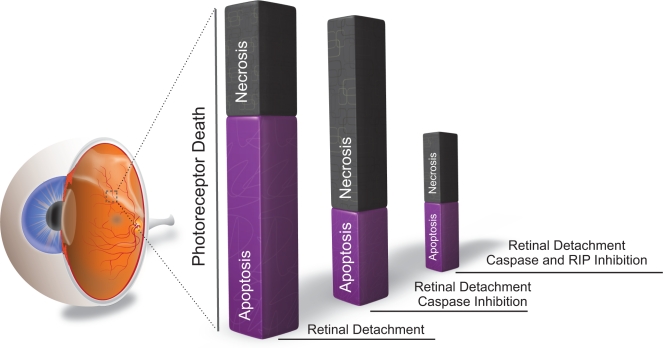
RIP kinase-mediated necrosis as a redundant mechanism of photoreceptor death Photoreceptor death is caused mainly by apoptosis after retinal detachment (left bar). Caspase inhibition by Z-VAD decreases apoptosis but promotes RIP kinase-mediated necrosis (middle bar). Blockade of both caspases and RIP kinases is required for effective prevention of photoreceptor loss (right bar).

During retinal degeneration, death ligands such as TNF-α and Fas-L are shown to be up-regulated and contribute to photoreceptor death [40,86]. As described above, death ligands can induce not only apoptosis but also necrosis. In addition, previous morphological analysis of photoreceptor death demonstrated the presence of both apoptosis and necrosis in retinal degeneration [60]. However, most of studies have not focused on necrosis since it was believed that necrosis is an unregulated form of cell death. In our recent work, we investigated the role of RIP kinase-mediated necrosis in experimental models of retinal detachment, and observed that RIP3 expression increases over 10-fold in the detached retina, especially in the outer nuclear layer. Morphological assessment revealed that necrotic photoreceptor death occurs after retinal detachment, although its frequency is approximately half that of apoptosis (Fig. 4A and D). Interestingly, treatment with the pan-caspase inhibitor Z-VAD decreases apoptosis but exacerbates necrosis after retinal detachment (Fig. 4B and D). The necrotic changes after caspase inhibition are reversed by co-treatment with Nec-1 or by genetic knockout of RIP3 (Fig. 4C and D)[64]. These data indicate that RIP kinase-mediated necrosis is an alternative pathway of photoreceptor death, which is utilized particularly when caspases are inhibited, and suggest that simultaneous inhibition of both RIP kinase and caspase pathways are necessary for effective prevention of photoreceptor death (Fig. 5). Since several death ligands are up-regulated during retinal degeneration [40,86], targeting the common downstream, i.e. RIP kinases and caspases, may be a useful strategy for preventing photoreceptor death mediated by various death signals.

One unexpected finding of our study is that RIP3 deficiency attenuates apoptotic photoreceptor death as well as necrosis after retinal detachment. In contrast, necrostatin-1 prevents only necrotic photoreceptor death without affecting apoptosis [64]. It remains unclear whether RIP3 signaling may affect not only RIP1 kinase-mediated necrotic pathway but also unknown apoptotic pathways. Previous studies reported that over-expression of RIP3 in cells leads to apoptosis induction [84,87]. Moreover, Upton and others recently demonstrated that mouse cytomegalovirus infection induces RIP3-dependent but RIP1-independent cell death in 3T3-Swiss albino fibroblasts [88]. These results suggest that RIP3 kinase may have additional substrates in addition to RIP1. Further biochemical and morphological analyses of RIP kinase-mediated cell death and identification of the direct substrates of RIP1 and RIP3 will further the characterization of RIP signaling pathways.

## DOWNSTREAM TARGETS OF RIP KINASES

### RIP and Reactive oxygen species

Previous studies in the 1990s reported that overproduction of reactive oxygen species (ROS) occurs in death receptor-mediated necrosis [79,89]. Consistent with these findings, recent studies have revealed the molecular links between RIP kinases and ROS-regulating enzymes. First, activated RIP3 interacts with metabolic enzymes such as glycogen phosphorylase (PYGL), glutamate-ammonia ligase (GLUL) and glutamate dehydrogenase 1 (GLUD1) [83]. PYGL catalyzes the degradation of glycogen to glucose-1-phosphate. GLUL and GLUD1 mediate glutaminolysis. GLUL catalyzes the synthesis of glutamine from glutamate and ammonia, and GLUD1 is a mitochondria matrix enzyme that converts glutamine to α-ketoglutarate. Activation of these enzymes eventually stimulates the Krebs cycle and oxidative phosphorylation, thereby increasing mitochondrial ROS production. Secondly, after TNF-α stimulation, RIP1 forms a complex with TNFR, Riboflavin kinase, and NADPH oxidase 1 [90,91]. NADPH oxidase is the best-characterized non-mitochondrial source of ROS and forms a membrane bound enzyme complex with p22^phox^ and Rac [92]. This complex generates superoxide by transferring an electron from NADPH in the cytsol to oxygen on the luminal side or in the extracellular space. Thirdly, RIP1 kinase activates autophagic degradation of catalase, which converts hydrogen peroxide to water and oxygen, thereby increasing ROS accumulation [93]. The requirement of ROS for RIP kinase-mediated necrosis has been demonstrated in several (albeit not all) types of cells [94]. In vivo, oxidative retinal damage after retinal detachment is suppressed by RIP kinase inhibition [64]. These findings suggest that ROS overproduction is an important downstream target of the RIP kinases during retinal detachment-induced photoreceptor necrosis. Oxidative stress has been implicated as a key mediator of photoreceptor death in various retinal diseases including age-related macular degeneration [95,96], retinitis pigmentosa [97] as well as retinal detachment [64,98]. Further studies will be required to address whether RIP kinases affect ROS production and photoreceptor death in a broad range of retinal disorders.

### RIP-mediated necrosis and Mitochondrial permeability transition

The release of mitochondiral proteins into the cytoplasm is a key event during cell death [99]. There are at least two distinct mechanisms for mitochondrial membrane permeabilization. First, mitochondrial outer membrane permeabilization (MOMP) is initiated by the formation of the Bax channel at the outer mitochondrial membrane, allowing for the release of cytochrom *c* and other intermembrane space proteins. Secondly, mitochondrial permeability transition (MPT) results from the opening of the permeability transition pore complex (PTPC), a polyprotein complex formed at the junction between the inner and outer mitochondrial membrane. The opening of PTPC leads to loss of the mitochondrial membrane potential, an influx of fluid into the matrix, swelling and rupturing of the outer mitochondrial membrane, and non-selective release of pro-death proteins [100]. It is postulated that apoptosis and necrosis may preferentially involve MOMP and MPT, respectively [101].

PTPC is a polyprotein complex primarily composed of voltage-dependent anion chanel (VDAC) in the outer membrane, adenine nucleotide translocator (ANT) in the inner membrane, and cyclophilin D in the matrix. Cyclophilin D is a critical component of PTPC formation, as genetic knockout of cyclophilin D prevents MPT and subsequent necrosis following H_2_O_2_ exposure or Ca^2+^ overload [102]. He and others recently reported that cyclophilin D-deficient MEFs are partially resistant to death receptor-mediated necrosis [82], suggesting that RIP kinase mediates necrosis through PTPC opening, at least in part. It is likely that MPT is important for photoreceptor death, as HIV protease inhibitors, which prevent PTPC opening, prevent cell death after retinal detachment [41,103]. In another study, RIP1 kinase was shown to inhibit the ANT function of transporting ADP into the mitochondria, resulting in reduced ATP and necrotic cell death [104]. Further studies of the molecular links between RIP kinase and PTPC components are required to elucidate the molecular signaling of necrosis.

### RIP and AIF

AIF is a flavoprotein, which, in the healthy state, is located in the mitochondrial intermembrane space and exerts a vital function in energy and redox metabolism [105]. However, under stress conditions, AIF is cleaved, translocates to the nucleus, and promotes chromatinolysis and cell death. AIF was first identified as a caspse-independent inducer of apoptosis [106], and recent studies showed that it also mediates programmed necrosis [28,107]. The translocation of AIF into the nucleus has been observed during photoreceptor death after retinal detachment and in inherited retinal degeneration [4,44,108]. Reduced AIF expression in *Harlequin* mutant mice reduces photoreceptor loss after retinal detachment [41]. Furthermore, our recent study showed that RIP kinase inhibition prevents AIF nuclear translocation after retinal detachment [64], suggesting the link between RIP kinase and AIF signaling. However, the precise molecular mechanisms by which RIP kinase regulates AIF translocation remain to be elucidated, as there are several steps between AIF translocation to cell death induction: processing of AIF in the intermembrane space [109], mitochondrial membrane permeabilization [110], and interaction with cyclophilin A for nuclear transport and chromatinolysis [111,112].

## CONCLUSIONS

Photoreceptor death in retinal degenerative disorders has been thought to be caused mainly by apoptosis. However, despite more than a decade of work on apoptosis, attempts to move drug-based neuroprotection for retinal degenerative diseases have failed [113]. Recent accumulating evidence identifies RIP kinase-mediated necrosis as an alternative pathway of cell death. In an experimental model of retinal detachment, we showed that, when caspase pathways are blocked, RIP kinase pathways promote photoreceptor necrosis and overcomes apoptosis inhibition. These findings suggest that photoreceptor death is redundantly regulated by apoptosis and necrosis, and that combined targeting of RIP kinases and caspases may provide effective neuroprotection in retinal disorders associated with photoreceptor loss.
